# Pre-Treatment Mutational and Transcriptomic Landscape of Responding Metastatic Melanoma Patients to Anti-PD1 Immunotherapy

**DOI:** 10.3390/cancers12071943

**Published:** 2020-07-17

**Authors:** Carol M. Amato, Jennifer D. Hintzsche, Keith Wells, Allison Applegate, Nicholas T. Gorden, Victoria M. Vorwald, Richard P. Tobin, Kelsey Nassar, Yiqun G. Shellman, Jihye Kim, Theresa M. Medina, Matthew Rioth, Karl D. Lewis, Martin D. McCarter, Rene Gonzalez, Aik-Choon Tan, William A. Robinson

**Affiliations:** 1 Department of Medicine, Division of Medical Oncology, University of Colorado Denver Anschutz Medical Campus, Aurora, CO 80045, USA; jhintzsche@gmail.com (J.D.H.); allison.applegate1106@gmail.com (A.A.); KELSEY.NASSAR@CUANSCHUTZ.EDU (K.N.); JIHYE.KIM@CUANSCHUTZ.EDU (J.K.); THERESA.MEDINA@CUANSCHUTZ.EDU (T.M.M.); MATTHEW.RIOTH@CUANSCHUTZ.EDU (M.R.); KARL.LEWIS@CUANSCHUTZ.EDU (K.D.L.); RENE.GONZALEZ@CUANSCHUTZ.EDU (R.G.); WILLIAM.ROBINSON@CUANSCHUTZ.EDU (W.A.R.); 2 Willamette Valley Cancer Institute and Research Center, Corvallis, OR 97330, USA; Keith.Wells@usoncology.com; 3 Saint Alphonsus Cancer Institute, Boise, ID 83706, USA; Nicholas.gorden@saintalphonsus.org; 4 Department of Surgery, Division of Surgical Oncology, University of Colorado Denver Anschutz Medical Campus, Aurora, CO 80045, USA; VICTORIA.VORWALD@CUANSCHUTZ.EDU (V.M.V.); RICHARD.TOBIN@CUANSCHUTZ.EDU (R.P.T.); MARTIN.MCCARTER@CUANSCHUTZ.EDU (M.D.M.); 5 Department of Dermatology, University of Colorado Denver Anschutz Medical Campus, Aurora, CO 80045, USA; YIQUN.SHELLMAN@CUANSCHUTZ.EDU; 6 Department of Biostatistics and Bioinformatics, Moffitt Cancer Center, Tampa, FL 33612, USA; AikChoon.Tan@moffitt.org

**Keywords:** immunotherapy, whole exome sequencing, melanoma, RNA sequencing, biomarker, anti-PD1, NFKB, CD83

## Abstract

Immunotherapy, such as anti-PD1, has improved the survival of patients with metastatic melanoma. However, predicting which patients will respond to immunotherapy remains a significant knowledge gap. In this study we analyzed pre-immunotherapy treated tumors from 52 patients with metastatic melanoma and monitored their response based on RECIST 1.1 criteria. The responders group contained 21 patients that had a complete or partial response, while the 31 non-responders had stable or progressive disease. Whole exome sequencing (WES) was used to identify biomarkers of anti-PD1 response from somatic mutations between the two groups. Variants in codons G34 and G41 in *NFKBIE*, a negative regulator of *NFkB*, were found exclusively in the responders. Mutations in *NKBIE*-related genes were also enriched in the responder group compared to the non-responders. Patients that harbored *NFKBIE*-related gene mutations also had a higher mutational burden, decreased tumor volume with treatment, and increased progression-free survival. RNA sequencing on a subset of tumor samples identified that CD83 was highly expressed in our responder group. Additionally, Gene Set Enrichment Analysis showed that the *TNFalpha* signaling via *NFkB* pathway was one of the top pathways with differential expression in responders vs. non-responders. In vitro *NFkB* activity assays indicated that the G34E variant caused loss-of-function of *NFKBIE*, and resulted in activation of *NFkB* signaling. Flow cytometry assays indicated that G34E variant was associated with upregulation of CD83 in human melanoma cell lines. These results suggest that *NFkB* activation and signaling in tumor cells contributes to a favorable anti-PD1 treatment response, and clinical screening to include aberrations in *NFkB*-related genes should be considered.

## 1. Introduction

Immune checkpoint blockade (ICB) treatments, such as anti-PD1, are now standard of care for patients with advanced metastatic melanoma [1,2,3,4]. Unlike targeted therapies, immunotherapies lack a clear genomic mutational hallmark, such as the *BRAF*^V600E^ mutation, that define response. Newer sequencing technologies allow for the analysis of tumor mutational burden or microsatellite instability that can be used to predict better response [5,6]. However, these measurements are often imprecise and are not sufficient to explain differences in response.

Recent whole genome and exome sequencing of melanoma tumors have uncovered other recurrent mutated genes besides the well-known *BRAF*, *NRAS,* or *KIT* somatic variants. For example, *SF3B1* mutations are frequently found in mucosal and ocular melanoma [7,8,9]. Mutations in *TERT*, *CDKN2A*, *NF1*, and *RAC1* are often mutated in cutaneous melanomas, and recurrent variants in *NFKBIE* are found in desmoplastic melanoma [7,10]. However, these studies often lack the clinical knowledge of patient outcomes such as treatment response, and thus do not address how the genomic variants delineates responsiveness to immunotherapy. 

Our study builds on previous reports that define the genomic and transcriptomic landscape of melanoma patient responses to immunotherapy. We employ a combination of whole exome sequencing, RNA sequencing, and in vitro analysis to characterize the pre-therapeutic landscape of metastatic melanoma in order to predict response to anti-PD1 therapy. Here we categorized melanoma patients as responders (complete and partial) or non-responders (stable disease and progressive disease) to anti-PD1 therapy. We present results from whole exome sequencing that identified recurrent mutations in *NFKBIE*, a negative regulator of *NFkB* that were found only in patients with a favorable response to anti-PD1 immunotherapy. RNA sequencing also showed that differential expression patterns exist between responders and non-responders, notably in the *TNFalpha/NFkB* signaling pathway. In vitro *NFKBIE* mutant variants resulted in activation of the *TNFalpha/NFkB* pathway and associated *TNFalpha*-induced expression of proteins involved with antigen presentation or co-stimulatory molecules. Together, these results suggest that activation of *NFkB* signaling in tumors enhances patient response to immunotherapy, and lack of a strong activation of this pathway may decrease response

## 2. Materials and Methods

### 2.1. Clinical Review

The Melanoma Biorepository at the University of Colorado Cancer Center currently contains over 4000 patient tissue and blood samples. A clinical review led to the identification of 52 patients with 71 total tissue samples that had previously undergone immunotherapy and had a pre-therapeutic biopsy (Table S1). Patients were then classified according to best response, defined using RECIST1.1 criteria [11]. Patient scans typically occurred at 3 and 6 months. In total, 21 patients were categorized as responders (5 complete responders, 16 partial responders) and 31 patients were classified as non-responders (16 with stable disease, and 15 with progressive disease). Formalin-fixed paraffin-embedded, and/or fresh frozen tissue samples then were assessed for quality and based on those results each sample underwent whole exome sequencing (WES) (71 samples), and/or RNA-sequencing (14 samples), Table S2. When available, normal peripheral blood DNA was also sequenced (whole exome) for mutational analysis.

### 2.2. Sample Collection and Nucleic Acid Isolation

All samples were collected between 2008 and 2016 stored in the Melanoma Biorepository at the University of Colorado. Informed written consent was obtained in accordance with approval from the Colorado Multiple Institutional Review Board and with the patient’s written consent (COMIRB-05-0309). Our institutional review board (the Colorado Multiple Institutional Review Board) allows us to consent and collect specimens, including archived specimens, from patients at any time. Therefore, the time interval between sample collection and subsequent treatment varied from years to months. Peripheral blood samples were collected in PAXGene (Qiagen, Valencia, CA, USA) DNA tubes and until processed were stored at 4 °C. Genomic DNA from tissues was isolated using either the DNeasy Blood and Tissue kit (Qiagen, Valencia, CA, USA) or the QiaAmp DNA formalin-fixed paraffin-embedded kit (Qiagen, Valencia, CA, USA) depending on the material. The concentration of DNA was determined using an ultraviolet spectrophotometer and stored at 4 °C. Total RNA was extracted from frozen tissue or cells using the RNeasy Plus Mini Kit (Qiagen, Valencia, CA, USA), with on-column DNase digest and tissue homogenization using the TissueLyser II (Qiagen, Valencia, CA, USA).

### 2.3. Next-Generation Whole-Exome Sequencing and Analysis

DNA concentration and purity were determined using Qubit (Thermo Fisher Scientific, Waltham, MA, USA) and Agilent 2100 Bioanalyzer (Agilent Technologies, Santa Clara, CA, USA) analysis. Genomic DNA (200 ng) was sheared using Covaris (Woburn, MA, USA) S220 at 150 bp. Sheared DNA was used to construct the exome library following Agilent SureSelect XT Target Enrichment System (Agilent Technologies, Santa Clara, CA, USA) for Illumina Paired End Multiplexed Sequencing Library (catalog #G9641B; Illumina, San Diego, CA, USA). Sheared DNA was end repaired, followed by addition of adapter tags to construct DNA libraries through PCR amplification. Exome capture was performed through hybridization using XT5 probe. Resulting captured libraries were indexed and purified. The cDNA library was validated on the Agilent 2100 Bioanalyzer using DNA-1000 chip. Libraries were sequenced on the Illumina HiSeq 2000 with 125 bp paired-end reads. We obtained an average of 400× and 200× sequencing coverage for the cancer and normal exomes, respectively. 

WES sequences were analyzed using our bioinformatics pipeline, IMPACT [12]. In brief, IMPACT is a discovery-oriented WES analysis pipeline designed to detect variant reads found in at least 10% of sequencing reads (minimum of four variant reads out of 20 mapped reads). To eliminate potential false positives, IMPACT performs a stringent filtering step, keeping only nonsynonymous somatic variants that are predicted to be deleterious by SIFT [13] and PolyPhen2 [14] or were present in the COSMIC database [15]. By doing this, IMPACT is able to detect more sub-clonal variants but only keeps those variants that are predicted to be the most deleterious. To remove germline variants, a normal blood DNA sample from the same patient was used as control when available. Somatic variants in genes were compared between responder and non-responder groups. A Fisher’s exact test was performed for each gene found in at least 30% of the samples. False discovery rate was applied to the gene list. Genes with a *p*-value less than 0.05 and false discovery rate less than 20% were considered for further analysis. Next generation sequencing data is deposited at the National Center for Biotechnology Information (Bethesda, MD, USA).

### 2.4. Next-Generation RNA Sequencing and Analysis

RNA library construction and sequencing were performed by the University of Colorado Cancer Center Genomics and Microarray Core. The Nugen Universal Plus mRNA seq kit was used for polyA selected RNA library construction. The libraries were sequenced as single-end reads at 150 cycle read lengths on the Illumina HiSeq2500 System. FASTQ files generated were processed using the Cufflinks/Cuffdiff RNAseq workflow and aligned to Ch37/hg19 [16]. Cuffdiff analysis generated fragment per kilobase per million mapped read (FPKM) values and performed differential gene expression analysis between sample groups. Differential gene expression and *p*-values were calculated using the Jensen–Shannon divergence method (JSD) and depicted as a heat map [17]. *p*-values were corrected for multiple testing and false discovery rates (FDR, *q*-values) using the Benjamini–Hochberg method, Table S3 [18]. Additionally, the fragment per kilobase per million (FPKM) values were analyzed by Gene Set Enrichment Analysis (GSEA, Broad Institute, v6.2MSigDB) to create a rank order gene list and identify pathways differentially utilized in the two sample groups [19]. Briefly, GSEA calculates and ranks the differential expression of genes, it further identifies pathways associated with those genes, and calculates a normalized enrichment score (NES) for a particular pathway. *p*-values (permutation method) were calculated and corrected for false discovery rates (*q*-values). For our analysis, the GSEA files included labels that assigned discrete phenotypes based on the clinical characteristics defined as responders and non-responders [20]. Collections used in this analysis include the HALLMARK gene sets from v6.2MSigDB [21]. 

### 2.5. Data Availability

The whole exome and whole transcriptome data have been deposited in NCBI’s Sequence Read Archive and Gene Expression Omnibus [22] and are accessible through SRA and GSE accessions: PRJNA639866 and GSE15996 (National Center for Biotechnology Information, Bethesda, MD, USA). 

### 2.6. NFKBIE Wildtype and G34E Expression Vectors

*NFKBIE* cDNA sequences were inserted into the pLVX-EF1a-IRES-ZsGreen1 vector (Clontech/Takara Bio, Mountain View, CA, USA, #631982) by GenScript using the SpeI/NotI restriction enzyme sites (Genscript, Piscataway, NJ, USA). Each construct was transformed into TOP10 competent *Escherichia coli* (*E. coli*) cells (Invitrogen, Carlsbad, CA, USA). The plasmids were purified using the HiSpeed Plasmid Midi kit (Qiagen, Valencia, CA, USA). 

### 2.7. NF-kB Luciferase Reporter Assay

HEK293t cells were cultured in DMEM media supplemented with 10% FBS, 1% penicillin/streptomycin, and 0.1 mM MEM non-essential amino acids (Invitrogen). In black, flat-bottomed tissue culture 96-well plates (Corning, Glendale, AZ, USA), *NFKBIE* expression vectors (0.4 ug), pGL4.32(*luc2P/*NF-kB-RE/Hygro) reporter vector (2.0 ug, Promega, Madison, WI, USA), and pRL-TK vector (0.2 ug, an internal transfection control, Promega) were transfected into HEK293t cells using Lipofectamine 2000 (Life Technologies). After 48 h, the cells were incubated with 10 ng/mL TNFalpha (Gemini Bio, Sacramento, CA, USA) for 4 h or media only. Reporter activity was measured using the Dual-Glo Luciferase assay system (Promega) and read on the Synergy 2 (BioTek, Winooski, VT, USA). 

### 2.8. Cell Surface Protein Analysis

Flow cytometry was performed on cells fixed with 1.0% paraformaldehyde and stained overnight in primary antibody (BioLegend, San Diego, CA, USA; PE anti-human CD83). Fluorescent intensity was measured using CytoflexS (Beckman Coulter, Indianapolis, IN, USA). 

## 3. Results

### 3.1. Clinical Analysis

We identified 52 patients with advanced (stage III unresectable and stage IV) metastatic melanoma who had tumor samples collected prior to undergoing immunotherapy, and with known clinical responses after immunotherapy. Sixty-four percent were male (34/52) with a median age of 56 years. Thirty-four patients had ctaneous melanoma (superficial spreading, nodular, or desmoplastic), and 8 patients (15%) had a non-sun exposed subtype. All patients had tumor samples taken before anti-PD1 immune checkpoint therapy of nivolumab (21%) or pembrolizumab (79%). According to RECIST1.1 criteria [11], 21 patients were categorized as responders (6 complete, 15 partial), and 31 were classified as non-responders (16 with stable disease and 15 with progressive disease). Approximately one-fifth of the patients (21%) had a tumor that was *BRAF*^V600E^ mutant. Our cohort has a low frequency of patients with *BRAF* mutations; likely a reflection that the majority of such patients being seen at our institution would have been offered targeted therapy. The WES patient cohort is summarized in Table 1. 

### 3.2. Mutated Genes Enriched in Patients that Responded to Immunotherapy Included the Hotspot Variant G34E in NFKBIE, (NFkB Inhibitor Epsilon) the Gene that Encodes for I-kappa-B-Epsilon (IkBe), a Negative Regulator of NFkB

**Figure 1:**
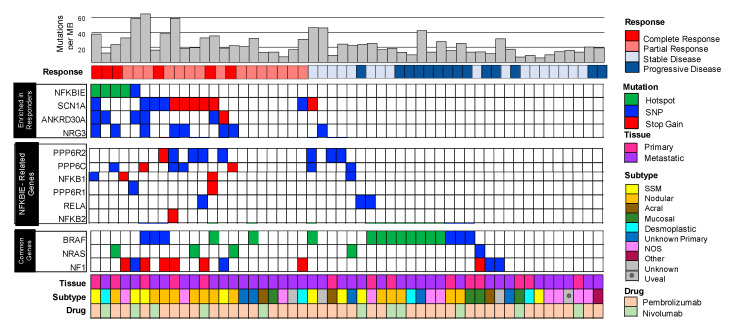
The pre-therapeutic mutational landscape of responders (*n* = 21) vs. non-responders (*n* = 31) to immunotherapy. Plot of clinical and genomic variants. Each column represents a separate patient. Each row represents, from top to bottom: mutational burden calculated as number of mutations per megabase, best response (RECIST criteria: from left to right complete and partial response; stable and progressive disease), gene variants enriched in responders (*NFKBIE*, *SCN1A*, *ANKRD30A*, and *NRG3*), variants in *NFKBIE*-related genes as identified by STRING v10, see methods (*PPP6R2*, *PPP6C*, *NFKB1*, *PPP6R1*, *RELA*, and *NFKB2*), common melanoma genes (*BRAF*, *NRAS*, and *NF1*). Patient clinical characteristics are described, from top to bottom as: type of tissue assayed (primary or metastatic lesion), primary type of melanoma (superficial spreading, nodular, acral, mucosal, desmoplastic, unknown primary, not otherwise specified, other, and unknown). Note in particular the genes enriched in responders including *NFKBIE* and *NFKBIE*-related genes. The common melanoma genes are evenly distributed between the groups.

The mutational landscape of pre-treated immunotherapy responders vs. non-responders is shown in Figure 1. Overall, 44 genes were found to be enriched (*p* < 0.05) in the responder group when compared to the non-responders group. *SCN1A* was the most significantly enriched gene and was mutated in 43% of responders compared to 6% of non-responders. *ANKRD30A* and *NRG3* mutations were also enriched in the responding group with 28% and 33% of samples mutated, respectively. However, these genes lack recurrent hotspot mutations, making it difficult to evaluate their biological function or clinical utility. Perhaps the most interesting gene found to be mutated and enriched in responders was *NFKBIE*, a negative regulator of *NFkB*. Here we detected recurrent hotspot mutations in 24% (5/21) of responders. Four of these patients harbored a G34R/E mutation while one harbored a G41E mutation (Figure S1). We decided to pursue *NFKBIE* and the hotspot variant G34E for potential clinical implications and effects on *NFkB* signaling. 

### 3.3. Enrichment of Mutations in NFKBIE-Related Genes Found in Responders Suggests Aberrations in the NFkB Signaling Pathway

*NFKBIE* is a negative regulator of the *NFkB* signaling cascade, a biological pathway which is important in cancer and inflammation [23,24]. In order to identify other potential mutants involved in this signaling cascade, the STRING (v10) database was used to identify other proteins that are known to interact with *NFKBIE* [25]. Six of these genes were also mutated more frequently in the responding cohort compared to the non-responders These genes along with *NFKBIE* comprise what we refer to here as the ‘*NFKBIE*-related genes’ and include: *NFkB1*, *NFkB2*, *RELA*, *PPP6C*, *PPP6R1*, and *PPP6R2*. In total, 67% (14/21) of responding patients had at least one mutation in one of the *NFKBIE*-related genes, compared to only 22% of non-responding patients (Figure 1). 

### 3.4. Mutations in NFKBIE-Related Genes Correlate with Clinical Metrics Used to Measure Patient Responses to Immunotherapy

**Figure 2:**
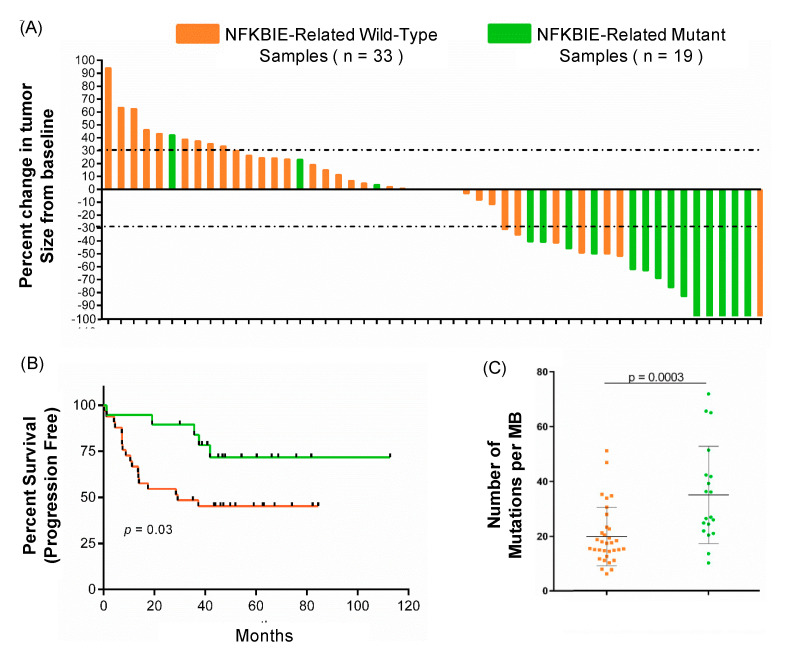
The clinical and genomic comparison of *NFKBIE*-related wild-type (*n* = 33) vs. mutant patients (*n* = 19). Patients with an *NFKBIE*-related gene mutation (either *PPP6R2*, *PPP6C*, *NFKB1*, *PPP6R1*, *RELA*, and *NFKB2*) are identified in green, and patients wild-type for these genes are identified in orange. (**A**) Waterfall plot depicting the percent change in tumor size by RECIST criteria. Fourteen patients with a mutation in a *NFKBIE*-related gene had tumor regression ≥ 30%, marked by grey dashed lines. (**B**) Progression free survival (PFS, *p* = 0.03) time in months for patients with and without a *NFKBIE*-related gene mutation. (Kaplan–Meier curves. *p*-values calculated using chi-squared, Mantel–Cox test). Patients harboring a tumor with a mutation in a *NFKBIE*-related gene had longer PFS times than patients who did not. (**C**) The difference in tumor mutational burden between tumors with a variant in a *NFKBIE*-related gene compared to those that are wildtype. Tumor mutational burden (TMB) is the measurement of the number of variants within the tumor as calculated by number of mutations per megabase as sequenced by whole exome, see methods (*p* = 0.003). The error bars represent standard deviation. *p*-values were calculated using an un-paired t-test.

To further understand the clinical and genomic impact of mutations in the *NFKBIE*-related genes, mutational burden, tumor volume, and patient survival were analyzed (Figure 2). Patients that respond to immunotherapy have been shown to have a higher mutational burden [5]. Our study confirmed those results (Figure S2) while also showing that patients with a mutation in *NFKBIE*-related genes have a higher mutational burden (Figure 2C, *p* = 0.003). The percent change of each patient’s sample was calculated from baseline. As demonstrated in the waterfall plot in Figure 2A, 84% (16/19) patients with a mutation in the *NFKBIE*-related genes had a decreased tumor volume after immunotherapy treatment. Of these 16 patients, 14 demonstrated a decreased tumor volume of over 30% (Figure 2A). Patients with *NFKBIE*-related gene mutations in addition to decreased tumor volume also demonstrated a significant increase in progression free survival (Figure 2B, *p* = 0.03). These data establish that patients with *NFKBIE* mutations have an overall higher mutational burden, decreased tumor volume, and a statistically significant increase in survival.

### 3.5. Gene Set Enrichment Analysis (GSEA) Suggests that Genes Involved in NFkB Signaling Are Differentially Expressed, Including Upregulation of CD83 in Our Responder Cohort

**Figure 3:**
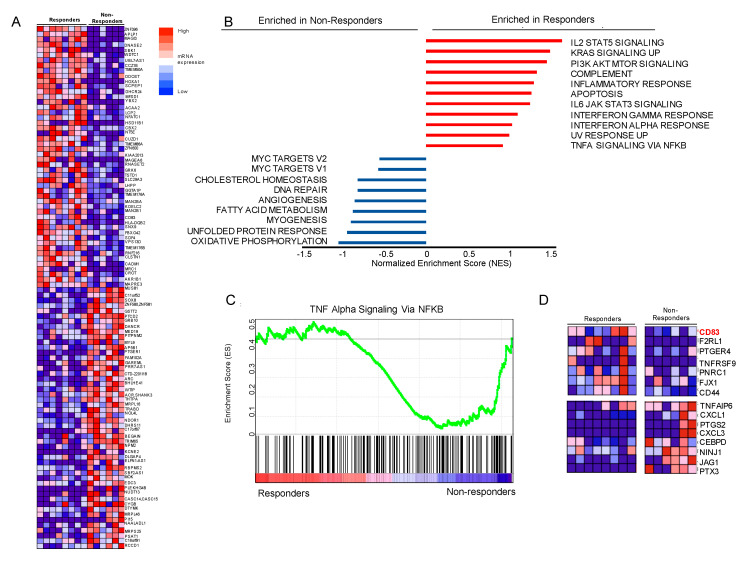
The pre-therapeutic transcriptional landscape of responders (*n* = 8) vs. non-responders (*n* = 6) to immunotherapy using Gene Set Enrichment Analysis (GSEA): The two outcome groups, responders and non-responders, were compared using GSEA (**A**) Heatmap of the expression profiles in patient tumors (columns) representing the 50 upregulated and 50 downregulated mRNA. The responders are on the left and non-responders are grouped on the right; the corresponding gene names are listed on the far-right side. Values represent gene expression calculated from the Jensen–Shannon divergence (JSD) method and are depicted as a range of colors: red being the highest to dark blue as the lowest. (**B**) The Hallmark pathway profiles of the two groups are graphed using their normalized enrichment score (NES). (**C**) Detailed enrichment plot of the Hallmark *TNFalpha* signaling via *NFkB* pathway. This depicts the following: *Top panel*-the running enrichment scores in green. *Bottom panel*—black vertical bars show position of genes within the ranked list (188 genes remain after filtering). (**D**) Emphasizing the ‘edge genes’ as a heatmap; the expression levels are represented for *CD83*, *F2RL1*, *PTGER4*, *TNFRSF9*, *PNRC1*, *FJX1*, and *CD44* (highly expressed in responders), and *TNFAIP6*, *CXCL1*, *PTGS2*, *CXCL3*, *CEBPD*, *NINJ1*, *JAG1*, and *PTX3* (downregulated in responders). Expression values and intensities are as described in (**A**).

Whole transcriptome analysis (RNAseq) was performed on the tumor samples that were available and that passed quality control. The RNA cohort of samples consisted of 8 responding tumor samples and 6 non-responding tumor samples. The heatmap (Figure 3A) identifies the 100 mRNAs differentially expressed between the responder and non-responder tumors. Expression values are visualized as dark red being the highest expressed mRNA to dark blue as the lowest. To identify biological states or processes that are involved in the two different patient therapeutic outcome groups, we performed GSEA using the Molecular Signatures Database Hallmark collection (MSigDBv6.2, methods). Figure 3B highlights biological processes. In this panel they are ranked by normalized enrichment score (NES), a statistic used to assess gene set enrichment results. Based on these criteria, we concentrated on the *TNFalpha* signaling via *NFkB* pathway for possible gene candidates to follow up (Figure 3C, Tables S3 and S4). Located at the left and right edges of the graph are *TNFalpha/NFkB* pathway genes most differentially expressed in our cohort. They are pictured as a heatmap in Figure 3D and include *CD83*, upregulated in responders and a gene of recent interest in oncology, (Figure S3) [26,27]. 

### 3.6. G34E of NFKBIE Is Likely a Loss-of-Function Variant, Resulting in Activation of NFkB Pathway

**Figure 4:**
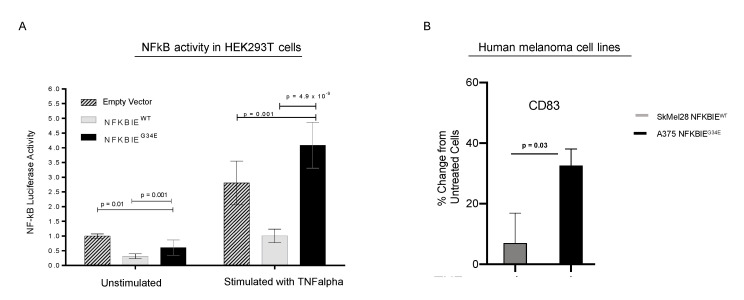
In vitro experiments to assess the effect of *NFKBIE* G34E on the NFKB pathway. Functional analysis of the *NFKBIE* G34E variant and wildtype was performed. Cells were stimulated with TNFalpha and monitored for NFkB activity via luciferase reporter assay or protein expression of CD83 by flow cytometry. (**A**) HEK293T cells were transiently transfected with a luciferase-based NFkB reporter, then either: empty vector or plasmids containing the *NFKBIE* G34E or *NFKBIE* wildtype cDNA sequences. Under TNFalpha stimulation, *NFKBIE* G34E transfected cells displayed an increase in luciferase activity when compared to cells transfected with empty vector (*p* = 0.001), and wildtype transfected cells (*p* = 4.9 × 10^−9^). When cultured without TNFalpha, transfected cells exhibited a decrease in luciferase activity. Values were normalized to empty vector in unstimulated conditions. (**B**) Flow cytometry analysis for CD83 in human melanoma cell lines that are *NFKBIE* wildtype and *NFKBIE* G34E mutant (SKMel28 and A375, respectively). Data are represented as the percent change in protein expression as compared to unstimulated cells. When stimulated, the A375 cell line showed a greater increase in CD83 protein expression as compared to SkMel28 (*p* = 0.03). For panels (**A**,**B**) all *p*-values were calculated using an un-paired t-test with Holm–Sidak correction for multiple tests. Data represent the standard deviation of the mean. The experiments were performed in technical triplicates, and independently repeated three times.

To determine how G34E affects *NFKBIE*’s inhibitory function on *NFkB* activity, we performed *NFkB* activity reporter assays using the HEK293T overexpression model (Figure 4A). Wildtype (WT), G34E *NFKBIE* cDNA plasmids, or empty vector (EV), with the luciferase-based *NFkB* reporter and normalizing plasmids were co-transfected into the cells, then incubated with and without TNFalpha ligand. We used luciferase as a readout for *NFkB* signaling and luminescent values were normalized to EV without stimulation. TNFalpha is a well-known activator of *NFkB*, while *NFKBIE* has been shown as a negative regulator of *NFkB* [28,29,30]. As expected, TNFalpha treatment induced *NFkB* activity drastically, and expressing WT *NFKBIE* blocked this induction, compared to EV. Moreover, expressing the G34E variant resulted in loss of *NFKBIE’s* inhibitory function, and significant activation of *NFkB*, as compared to cells transfected with WT, *p* = 4.9 × 10^−9^. G34E transfected cells even displayed higher *NFkB* activity than cells with EV (*p* = 0.001). At the basal level without TNFalpha stimulation, G34E cells also displayed slightly higher luciferase activity compared to the WT, further indicating that G34E is a loss-of-function variant. Consistent with the idea that CD83 is downstream of *NFkB* and can be affected by *NFKBIE* variants in melanomas, human melanoma cell line with G34E variant, A375, displayed TNFalpha-induced CD83 expression, compared to melanoma cell line with WT variant, SKEML28, (Figure 4B, Figure S4). Other downstream targets of *NFkB*, such as PDL1 and major histocompatability complex (MHC) class II, were also examined (Figure S4). However, the presence of these molecules was not particularly striking.

## 4. Discussion

Understanding how tumors evade the immune system marked a major turning point in the treatment of patients with metastatic melanoma and other cancers. With the use of single agent anti-PD1, 40–50% of patients with advanced or metastatic melanoma will have an initial response to therapy, and overall, 30–40% of patients will have a long-term durable clinical benefit [4]. However, the intersection of immunology and cancer biology creates challenges whereby a single genomic driver or biomarker (for example *BRAF*, *KIT*, or *EGFR* mutations in targeted therapies) do not adequately explain or predict response among patients being treated with anti-PD1 therapies. Therefore, it is important to explore mutational events that could predict favorable outcomes to anti-PD1 therapies. 

Previous large-scale next generation sequencing studies of patients with melanoma undergoing immune checkpoint blockade have not fully investigated pre-therapeutic somatic mutations as predictors of response. Hugo et al. found that *BRCA2* mutations were enriched in melanomas responsive to anti-PD1. These authors however did not link specific genomic variants with mRNA expression signatures in their transcriptome analysis [31]. Gide et al. combined RNAseq plus multiplex proteomics to analyze patients undergoing anti-PD1 monotherapy or in combination with anti-CTLA4. Their study focused on immune cell phenotyping and identified that EOMES + CD69 + CD45RO + T cells are associated with response [32]. In a large cohort of patients with metastatic melanoma, Liu et al. also analyzed pre-immune checkpoint blockade treated tumors using whole exome and whole transcriptome sequencing and suggested that MHC class I and II may predict response [33]. Interestingly, expression of MHC class I and class II molecules can be influenced by several factors including *INFgamma*, *TNFalpha*, and *NFkB* signaling [34,35]. Although they did not highlight any individual gene or variant that was significantly associated with response, their data support our finding. For example, the *NFKBIE* G34E variant and variants in related genes, such as *PPP6R2*, *PPP6C*, *PPP6R1*, *RELA*, *NFKB2*, and *NFKB1*, were found in several patients classified as complete or partial responders in the Liu et al. cohort. These are listed in the Liu et al. supplemental tables [33]. In a study similar to ours, Yu et al. used whole exome and transcriptome analysis of non-cutaneous melanoma and showed that *CDK4* gene copy-number altered the expression of genes in *TNFalpha/NFkB* and *INFgamma* signaling pathways. It is unclear if they identified variants in *NFKBIE*-related genes; however, several cell cycle molecules are regulated by *NFkB* transcription [36,37]. We had too few non-cutaneous melanomas in our cohort to confirm their findings. 

Our findings recapitulate current trends in which the average tumor mutational burden (TMB) is higher in the responder group compared to non-responders. Tumor mutational burden is commonly used as a predictor for immunotherapy response, yet it has weaknesses. In a review by Chan et al., one challenge of using this metric is that it “assigns equal weight to each tumor mutation” and ignores the need for integrating variants into treatment responses. Furthermore, patients with low TMB can respond to checkpoint inhibition suggesting that some mutations may be more important than others. In this study, we identified specific somatic mutations in *NFKBIE* with recurrent hotspot locations at codons G34 and G41, among patients who responded favorably to immunotherapy. By exploring other proteins that interact with *NFKBIE*, we also identified other mutations in *NFKBIE*-related genes that occurred more frequently in patients who responded positively to immunotherapy. Here, we show that samples with *NFKBIE*-related gene mutations had a significantly higher mutational burden than the wild-type cohort. High tumor mutational burden is often observed in cancers with deficiencies in genes involved with DNA damage response [38]. However, in this present study we cannot conclude if these mutations play a role in DNA damage response mechanisms. Patients with *NFKBIE*-related gene alterations also had significant clinical differences, including a greater shrinkage in tumor volume and a significant increase in progression free survival. Moreover, our study went beyond just sequencing the tumor which includes immune cells and the tumor microenvironment, and experimenting with melanoma cells. Our results indicated that the pathways we identified could be important within melanoma tumor cells, not immune cells. These data emphasized the potential functional impact the mutations caused in the melanoma itself that could ultimatley impact how the immune system responds. 

*NFKBIE* is well described as a negative regulator of *NFkB* and *NFKBIE* has at least two isoforms, as described below. The genomic coordinates for the variants at codon G34 (chr6:44,233,400) are the same described by two separate genome sequencing studies in melanoma [7,39]. These studies did not address treatment outcomes in their patient cohorts. In desmoplastic melanoma, Shain et al. identified 20 clustered *NFKBIE* mutations in 15 of their tumor samples (across their study the overall frequency was 14.5%). The authors postulated two possible models for these *NFKBIE* variants—either they reside in the coding region of the long isoform or in the promoter region of the short isoform [39]. The G34E variant is in the coding region of the long isoform, and our in vitro data show that this mutation is loss-of-function, resulting in increased *NFkB* activity; as discussed below. Recent reports indicate that patients with desmoplastic melanomas have an overall response rate of 70% to immunotherapy [40]. Interestingly, our cohort contains one patient with desmoplastic melanoma who harbored a *NFKBIE* G34E mutation and had a complete response to immunotherapy. Further studies are needed to determine if this outcome would be similar in a larger desmoplastic melanoma patient set. 

Our study confirms similar reports about the effect of *NFKBIE* variants on *NFkB* activity in rheumatoid arthritis (RA) and lymphoid malignancies (chronic lymphocytic leukemia (CLL), primary mediastinal B-cell lymphoma (PMBL) and Hodgkin lymphoma (HL)) [41,42,43]. In our overexpression experiments, *NFkB* activity was higher in the *NFKBIE* G34E transfected cells at both baseline and upon stimulation. The decrease in luciferase activity seen in stimulated and unstimulated wildtype transfected cells reflects the inhibitory nature of *NFKBIE* on *NFkB* signaling [29,30]. Similarly, non-synonymous single nucleotide polymorphisms in *NFKBIE* (6p21.1, rs2233433, and rs2233434) seen in patients with RA, leads to enhanced *NFkB* activity [43]. Likewise, in CLL, *NFKBIE* aberrations leads to functional loss of the protein it encodes, IkBe, resulting in nuclear translocation of RELA [42]. For these patients, *NFkB* signaling contributes to a worse outcome. However, our studies suggest the effects of *NFKBIE* variants may be from tumor cells, which are different from the RA or lymphoid malignancies. 

As a transcription factor, *NFkB* modulates mRNA (and by extension protein) expression [44]. We speculated that these downstream targets ultimately interact with several diverse cellular pathways, particularly processes involved with immune function and inflammation [24,45]. To further investigate this, we examined the transcriptome of available patient tumors. Using Gene Set Enrichment Analysis (GSEA), we showed that tumors from responding patients in our cohort had enrichment of genes related to immune and inflammatory responses. Consistent with previous reports of patients with melanoma, our transcriptomic data also showed several differentially expressed genes involved with the *IFNgamma* and *Interleukin/STAT* signaling pathways [31,32,33,46]. Similar to Liu et al., we saw higher expression of MHC class II molecules in responders [33]. However, unique in the current study is the finding of enrichment of genes involved in *TNFalpha/NFkB* signaling. Of particular interest was *CD83*, a member of the immunoglobulin (Ig) superfamily, which is overexpressed in responders. 

CD83 is a marker of mature dendritic cells and involved in the development of B and T cells. It has both soluble and membrane forms, and is emerging as an interesting immune-modulating molecule [47,48,49]. Prior studies have shown that *CD83* is a downstream target of *NFkB*, and can be detected on solid and hematologic malignancies, and has been shown to influence the tumor microenvironment [50,51,52,53,54,55]. In patients with Hodgkin lymphoma, it can be secreted from tumor cells to influence expression of PD1 in surrounding T cells [26], and there are promising results using nivolumab in Hodgkin lymphoma patients [56,57]. Our study supports a role for *CD83* in patients receiving anti-PD1 therapy. We propose that activation of *NFkB* signaling may lead to expression of *CD83* at the mRNA and protein level. To expand on these findings, future studies are planned that would include the use of advanced techniques such as single cell RNA-seq or multiplex immunohistochemistry.

## 5. Conculusions

In summary, this study is the first to identify recurrent mutations in *NFKBIE*, a negative regulator of *NFkB*, as a partial genomic explanation for response to anti-PD1 immunotherapy in melanoma patients. Pre-treatment *NFKBIE* and *NFKBIE*-related gene mutations in patient samples correlated with clinical outcomes including decreased tumor volume and progression free survival. Additionally, by demonstrating that loss-of-function variants in *NFKBIE* activates *NFkB* signaling in tumor cells, and by identifying differentially expressed genes involved in *TNFalpha/NFkB* signaling in our cohort, we are able to show that this may be an important biological pathway in melanoma patients undergoing anti-PD1 therapy. The clinical, genomic, transcriptomic, and in vitro data presented indicate that increased tumor cell *TNFalpha/NFkB* signaling, as a consequence of an *NFKBIE* mutation, may contribute to a favorable anti-PD1 treatment response.

## Figures and Tables

**Table 1:** Cohort clinical characteristics.

Total Cohort	52
**Sex**	
Male	34 (65%)
Female	18 (35%)
**Age (year)**	
Median	56
Range	22–87
**Mutational Analysis**	
BRAF (V600E/K)	11 (21%)
NRAS (G13_/Q61_)	4 (7%)
NF1	11 (21%)
**Immune Checkpoint Therapy**	
Nivolomab	11 (21%)
Pembrolizumab	41 (79%)
**Response**	
Complete Response	6 (11%)
Partial Response	15 (28%)
Stable Disease	16 (30%)
Progressive Disease	15 (28%)
**Melanoma Subtype**	
Superficial Spreading	17 (33%)
Nodular	13 (25%)
Desmoplastic	4 (7%)
Unknown Primary	5 (9%)
Acral	3 (5%)
Mucosal	4 (7%)
Ocular	1 (2%)
Other	5 (9%)
**Stage at Diagnosis**	
III Unresectable	1 (2%)
IIIB	2 (3%)
IIIC	10 (19%)
IV	39 (75%)

## Supplementary Materials

The following are available online at https://www.mdpi.com/2072-6694/12/7/1943/s1, Figure S1. Validation of *NFKBIE* hotspot variants by PCR and Sanger sequencing; Integrative genomics viewer of patient tumors for Chr6:44,233,400, Figure S2. Tumor mutational burden in responders and non-responders to anti-PD, Figure S3. qRT-PCR validation of *CD83* mRNA in tumor samples from responders and non-responders to anti-PD1, Figure S4. Downstream targets of *NFkB* including PDL1 and MHC class II proteins in *NFKBIE* G34E mutated specimens, Table S1. Patient clinical characteristics, Table S2. Specimen overview and analysis, Table S3. Gene Set Enrichment Analysis; HALLMARK Pathway Enrichment For the Cohort, Table S4. Gene Set Enrichment Analysis; Hallmark TNFalpha_signaling_via_NFkB Gene Enrichment Scores.

## Abbreviations

Ankyrin Repeat Domain 30A(ANKRD30A); B-Raf Proto-Oncogene(BRAF); BRCA2 DNA Repair Associated(BRCA2); CD44 Molecule(CD44); CD83 Molecule(CD83); Cyclin Dependent Kinase 4(CDK4); Cyclin Dependent Kinase Inhibitor 2A(CDKN2A); CCAAT Enhancer Binding Protein Delta(CEBPD); Cytotoxic T-Lymphocyte Associated Protein 4(CTLA4); C-X-C Motif Chemokine Ligand 1(CXCL1); C-X-C Motif Chemokine Ligand 3(CXCL3); Epidermal Growth Factor Receptor(EGFR); F2R Like Trypsin Receptor 1(F2RL1); Four-Jointed Box Kinase 1(FJX1); Interferon Gamma(INFgamma); Jagged Canonical Notch Ligand 1(JAG1); KIT Proto-Oncogene(KIT); Neurofibromin 1(NF1); Nuclear Factor Kappa B(NFkB); Nuclear Factor Kappa B Subunit 1(NFKB1); Nuclear Factor Kappa B Subunit 2(NFKB2); NFKB Inhibitor Epsilon(NFKBIE); Ninjurin 1(NINJ1); NRAS Proto-Oncogene(NRAS); Neuregulin 3(NRG3); Programmed Cell Death 1(PD1); Programmed death ligand 1(PDL1); Proline Rich Nuclear Receptor Coactivator 1(PNRC1); Protein Phosphatase 6 Catalytic Subunit(PPP6C); Protein Phosphatase 6 Regulatory Subunit 1(PPP6R1); Protein Phosphatase 6 Regulatory Subunit 2(PPP6R2); Prostaglandin E Receptor 4(PTGER4); Prostaglandin-Endoperoxide Synthase 2(PTGS2); Pentraxin 3(PTX3); Rac Family Small GTPase 1(RAC1); Response Evaluation Criteria in Solid Tumours(RECIST); RELA Proto-Oncogene(RELA); Sodium Voltage-Gated Channel Alpha Subunit 1(SCN1A); Splicing Factor 3b Subunit 1(SF3B1); Telomerase Reverse Transcriptase(TERT); Tumor Necrosis Factor alpha(TNFA); TNF Alpha Induced Protein 6(TNFAIP6); TNF Receptor Superfamily Member 9(TNFRSF9).
