# Supplementary material for: Pre-Treatment Mutational and Transcriptomic Landscape of Responding Metastatic Melanoma Patients to Anti-PD1 Immunotherapy
Source: Cancers (Basel). 2020 Jul 17;12(7):1943. doi: 10.3390/cancers12071943 (PMC7409244; doi:10.3390/cancers12071943)
Supplement: Supplementary file 1 [file cancers-12-01943-s001.zip › cancers-836356-supplemenraty-final/cancers-836356 - supplementary.docx]

Supplementary Materials


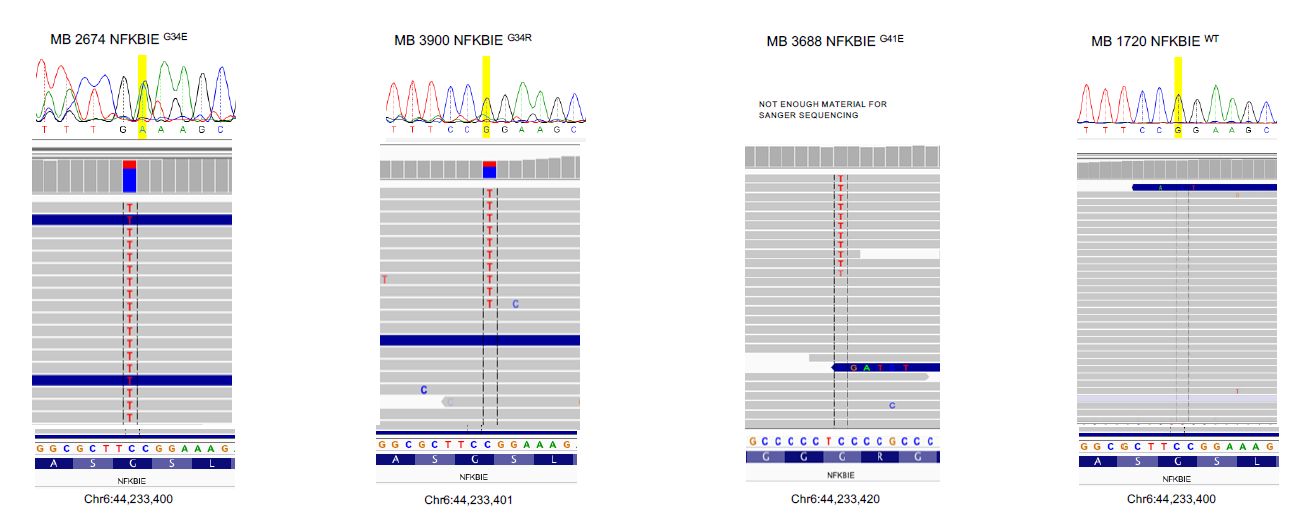


**Figure S1.** Validation of NFKBIE hotspot variants by PCR and Sanger sequencing. Also depicted are IGV images.


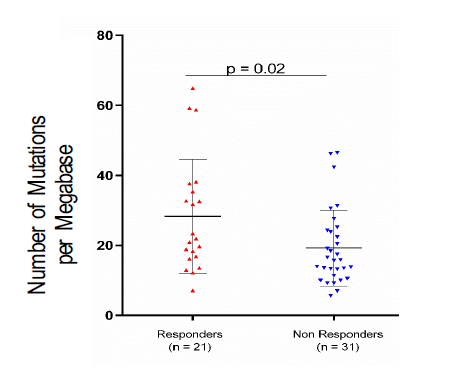


**Figure S2.** Tumor mutational burden in responders and non-responders.


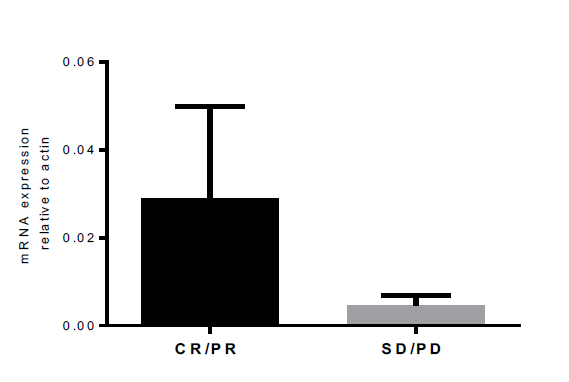


**Figure S3.** qRT-PCR validation of CD83 mRNA in tumor samples from responders and non-responders to anti-PD1.


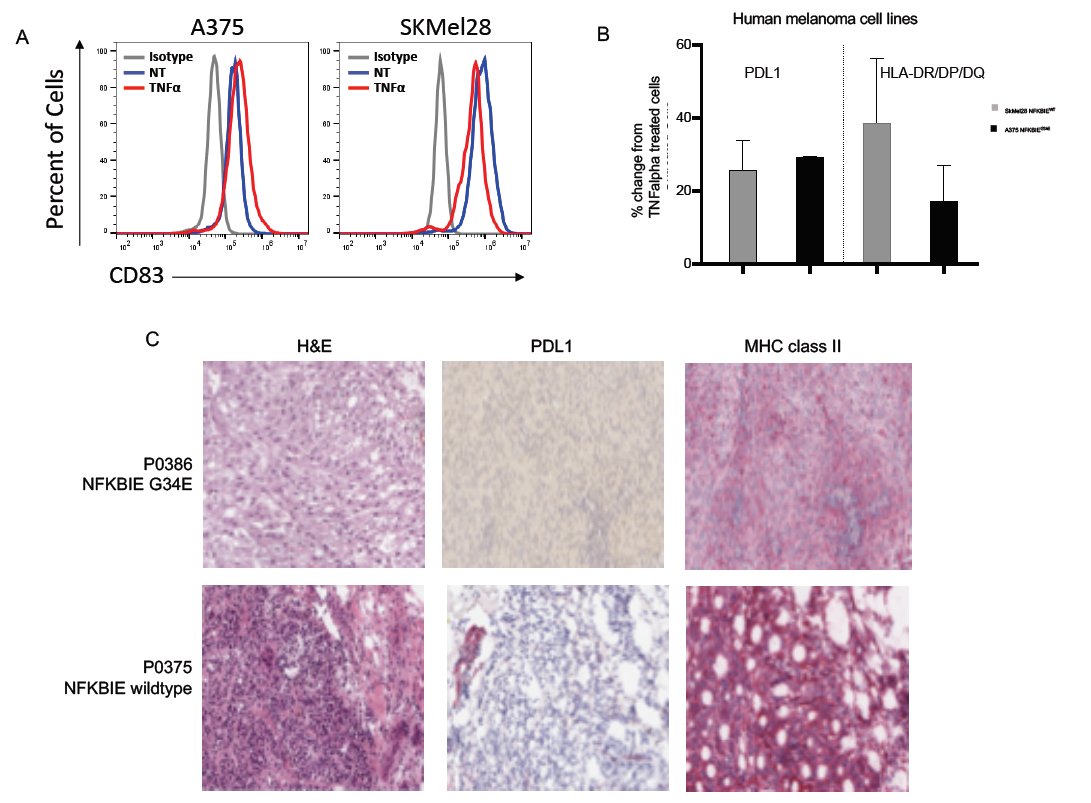


**Figure S4.** Downstream proteins CD83, PDL1, and MHC class II proteins, were assayed in NFKBIE G34E mutated specimens. (**A**). Comparisons of CD83 expression following stimulation with TNFalpha. (**A**) Histograms comparing the expression of CD83 between untreated (NT) cells or TNFalpha stimulation in A375 or SKMel28 cell lines. (**B**) Human melanoma cell lines A375 and SKMEL28 assayed for PDL1 and HLA-DR/DP/DQ by flow cytometry when treated with TNFalpha. (**C**) Immunohistochemistry staining of tumors from patients NFKBIE G34E and wildtype tumors.
